# A pharmacovigilance study on probiotic preparations based on the FDA Adverse Event Reporting System from 2005 to 2023

**DOI:** 10.3389/fcimb.2025.1455735

**Published:** 2025-05-13

**Authors:** Yitong Wang, Weifu Tan, Xuyang Li, Guangli Yang, Yunxiao Wang, Jing Liao, Aner Lu, Guoqing Zhang, Kuidai Chen, Liling Yang, Wei Li

**Affiliations:** ^1^ Department of Neonatology, Binhaiwan Central Hospital of Dongguan, Dongguan, China; ^2^ Department of Pediatric, The First Clinical Medical College of Jinan University, The First Affiliated Hospital of Jinan University, Guangzhou, China; ^3^ Department of Central Laboratory, Binhaiwan Central Hospital of Dongguan, Dongguan, China; ^4^ Dongguan Key Laboratory of Prevention and Treatment of Critical Illness, Binhaiwan Central Hospital of Dongguan, Dongguan, China

**Keywords:** probiotic, FAERS, pharmacovigilance, adverse events, adverse drug reactions, gender, age

## Abstract

**Background:**

Probiotics are recognized as beneficial foods, but adverse reactions reported by individuals still exist. This study aims to analysis adverse events (AE) related to probiotics from the FAERS database from the first quarter (Q1) of 2005 to the fourth quarter (Q4) of 2023.

**Methods:**

The AE data related to probiotic from the 2005 Q1 to the 2023 Q4 were collected. R language was applied to analyze the standardized AE data and three algorithms including the reporting odds ratio (ROR), the proportional reporting ratio (PRR) and the empirical Bayes geometric mean (EBGM) were used to identify AE signals.

**Results:**

In this study, 10,698,312 reports were collected from the FAERS database, of which 74 probiotic-related adverse events were reported. About one third of the reported cases were older than 60 years.36.36% of the reported cases required Hospitalization. A total of 285 preference terms (PTS) and 15 system organ classes (SOC) were identified. In the overall analysis, only 9 PTs and 2 SOCs met significant disproportionality for all three algorithms simultaneously. SOCs included Gastrointestinal disorders (N=97, ROR=5.3, PRR=3.84, EBGM=3.84) and Hepatobiliary disorders (N=9, ROR =3.39, PRR=3.32, EBGM=3.32). PTs included Gastrointestinal pain (ROR=77.76, PRR=76.69, EBGM=76.63), Hypophagia (ROR=24.13, PRR=23.88, EBGM=28.88), and Hepatobiliary disorders (N=97, ROR=5.3, PRR=3.84, EBGM=3.84) and Flatulence (ROR=23.75, PRR=23.28, EBGM=23.27) were the top four highest. Meanwhile, s found new unique adverse signals such as Agitation (ROR=12.48, PRR=12.32, EBGM=12.32) and Anxiety (ROR=4.10, PRR=4.04, EBGM=4.04). Additionally, subgroup analyses were performed to identify AE signals based on gender and age. Metabolism and nutrition disorders (N=6, ROR=3.21, PRR=3.04, EBGM=3.04) and Asthenia (N=3, ROR=5.9, PRR=5.71, EBGM=5.71) were unique AE signal for the male group.

**Conclusion:**

Although, the risk of adverse reactions arising from the application of probiotics cannot be ignored. However, However, the results of this FAERS-based study continue to support the overall safety of probiotic preparations. It is necessary to pay attention to the potential influence of factors such as gender and age on the effects and adverse reactions of probiotic application in basic research and clinical application.

## Introduction

Probiotics are recognized as a class of living microorganisms that, when based on adequate dosage, can have beneficial effects on the health of the host (Hill et al., 2014). It has also been suggested that probiotics, as a class of bacteria that live in the human gut and are involved in metabolism and immune function, can be regarded as metabolic “organs” (Chua et al., 2017). The main probiotics that are widely used today are *Lactobacillus*, *Bifidobacterium*, specific *Escherichia coli*, *Enterococcus faecium*, *Pediococcus*, and *Yeast* species, as well as other intestinal commensals, which are mainly Gram-positive bacteria (Mazziotta et al., 2023). These applications of probiotic have also benefited from the growth-driving effect of advances in clinical efficacy measurement of probiotic-based products over the last two decades.

Probiotics have a variety of usefulness. Oral probiotics are one of the oldest forms of microbial therapy and are effective not only in preventing and reducing the severity of acute diarrhea in children, but also in alleviating antibiotic-associated diarrhea and have even been used to reduce certain allergies in children (Macfarlane and Cummings, 2002; Tan et al., 2021). In addition, probiotic interventions have been proposed to improve skin health as well as treat certain eye diseases (Chisari et al., 2017; Gao et al., 2023). Probiotics can affect central nervous system-related functions by influencing the gut microbiota, a process known as the “microbiota-gut-brain axis” (Fan et al., 2023). Probiotic transplants can alleviate overeating disorders by restoring the gut microbiota and gut metabolic environment and restoring normal activity of the brain-gut axis. Oral probiotic vaccines induce intestinal mucosal immunity, and the probiotics themselves are capable of producing metabolites with anti-inflammatory cytokine effects, which holds promise for intestinal cancer prevention (Singh et al., 2022).

The widespread consumption of probiotics by the general public has led to a dogma that probiotics are beneficial to the general population (Abid and Koh, 2019). The early days of probiotic application, when they were not considered as drugs, may also have led to the neglect of monitoring and reporting of probiotic adverse events (AEs) by the public community (Merenstein et al., 2023). A review by Agency for Healthcare Research and Quality (AHRQ) reported that although the available evidence from clinical studies does not support that the application of probiotics will increase health risks, the research literature has largely neglected to adequately assess and report on safety (Hempel et al., 2011). However, AEs of consuming probiotics exist in both theory and practice. Although most of the evidence supports the hypothesis that probiotics are generally safe for the majority of the population, some studies have identified theoretical risks, including serious infections, harmful metabolic activity, immune stress and gastrointestinal dysfunction in susceptible individuals (Doron and Snydman, 2015). Consumption of *L. rhamnosus GG* may be followed by sepsis, especially in high-risk groups (Salminen et al., 2004; Honeycutt et al., 2007). Studies have shown that the microbiota, including probiotics, can influence the efficacy of drugs through chemical transformation, even if this process is not what the host wants to happen (Koppel et al., 2017). Therefore, the identification of probiotic-associated adverse reaction signals cannot be ignored, which is an important guide for clinical application.

The FDA Adverse Event Reporting System (FAERS) is the most influential national-level AE reporting monitoring database in the world and is one of the primary tools for pharmacovigilance today (Alomar et al., 2020). This database is used for FDA’s post-market safety monitoring of all marketed drugs and therapeutic biologic products and is an extensive collection of reports of AEs received from manufacturers, consumers, and healthcare professionals. Currently, there is still a lack of systematic and comprehensive adverse drug reactions (ADR) studies associated with probiotic preparations based on real-world and big data. Therefore, this study intends to dig deeper into the data in the FAERS database and statistically analyze the real-world adverse reaction signals of probiotic preparations from different perspectives in order to obtain more reliable results.

## Method

### Data sources

The FAERS database integrates millions of AEs and related data reported by healthcare professionals, drug manufacturers, and others. This database has been updated quarterly since 2004 and continues to be freely available to the global public (Zhou and Hultgren, 2020). In the current study, we downloaded data on relevant AEs associated with probiotic preparations from the FAERS database for the period from the first quarter (Q1) of 2005 through the fourth quarter (Q4) of 2023.

### Cleaning and standardization of drug names and adverse drug reactions

Data downloaded from the FAERS database were cleaned using the R language to collect clean and standardized data by removing duplicate reported data. In this study, we used probiotics as the primary suspect (PS). In addition, the latest version of the Medical Dictionary of Regulatory Activities (MedDRA; version 25.1) was used to match the preferred terms (PTS) for probiotic adverse reactions. It is essential to note that MedDRA has five levels from low to high: the lowest-level term (LLT), preferred term (PT), high-level term (HLT), high-level group term (HLGT), and system organ class (SOC) (Tang et al., 2024). We choose to list the SOCs that corresponded to these PTSs. We collected clinical characteristics of patients with adverse events associated with probiotic dietary supplements, such as gender, age, reporting region, reporter, time of reporting, and outcome.

### Signal mining

**Figure 1:**
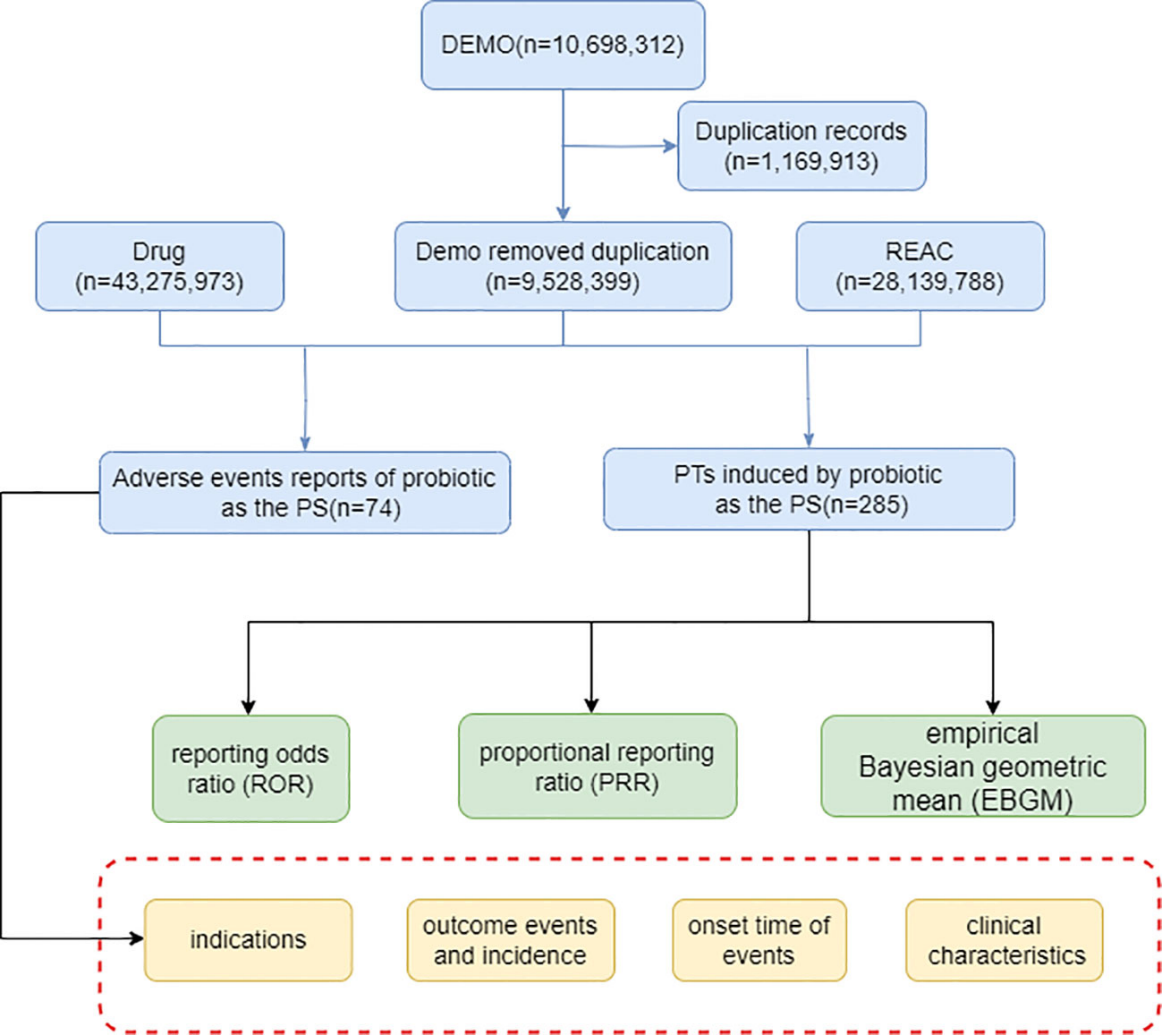
The flow diagram of selecting probiotic-related AEs from FAERS database.

A key component of detecting safety signals in marketed medicinal products is the collection and evaluation of individual case reports, of which disproportionality analysis remains the predominant assessment tool. It is based on the comparison between the number of theoretical reports and the number of reports actually observed in a combination of suspected drugs and AEs (Caster et al., 2020). Disproportionality is usually measured using a four-grid scale (**Supplementary Table S1**) and used this table as the basis for the subsequent calculation. The reporting odds ratio (ROR) compares the odds of reporting AEs associated with the interest target drug to all other events (Rothman et al., 2004), and we choose ROR to detect signals of adverse events in probiotics reports in this study. We also used proportional reporting ratio (PRR) and the empirical Bayesian geometric mean (EBGM) method, another method of detecting potential associations between reported probiotics and adverse reactions (Evans et al., 2001; Slade et al., 2009). The three algorithms specific formulas and positive signal selection criteria were shown in **Table 1**. Finally, results that met the positive signal selection criteria of the three algorithms above were considered valid ADR. All data analysis in this study was realized through R studio, and the chi-square test was used for intergroup comparisons. The overall analytical flow of this study is shown in **Figure 1**.

**Table 1:** Three algorithms specific formulas for signal detection.

Algorithms	Formula	Criteria
ROR	$ROR=\frac{a\times b}{c\times d}$	*N*≥3, 95%*CI* ≥ 1
	$95\%CI=e^{\ln(ROR)\pm 1.96\sqrt{\left(\frac{1}{a}+\frac{1}{b}+\frac{1}{c}+\frac{1}{d}\right)}}$	
PRR	$PRR=\frac{a(c+d)}{c(a+b)}$	*N*≥3, PRR≥2, *χ2* ≥ 4
	$\chi^{2}=\frac{(a+d+c+d){\left(ad-bc\right)}^{2}}{\left(a+b)(c+d)(a+c)(b+d\right)}$	
EBGM	$EBGM=\frac{a(d+b+c+d)}{\left(a+c)/(a+b\right)}$	EBGM05 > 2
	$95\%CI=e^{\ln(EBGM)\pm 1.96\sqrt{\left(\frac{1}{a}+\frac{1}{b}+\frac{1}{c}+\frac{1}{d}\right)}}$	

a, number of reports containing both the target drug and target adverse drug reaction; b, number of reports containing other adverse drug reaction of the target drug; c, number of reports containing the target adverse drug reaction of other drugs; d, number of reports containing other drugs and other adverse drug reactions; N, number of reports; 95%*CI*, 95% confidence interval; *χ2*, chi-squared; EBGM05, the lower bound of 95% *CI.*

## Result

### Basic information about AEs of probiotic

**Figure 2:**
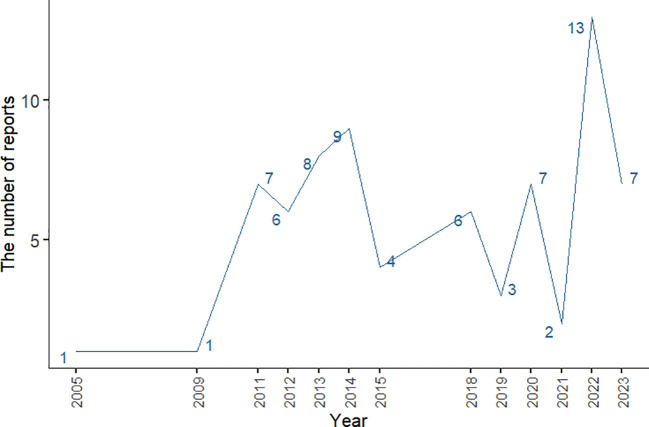
Line graph of changes in the number of annual case reports of AE associated with probiotic preparations.

**Figure 3:**
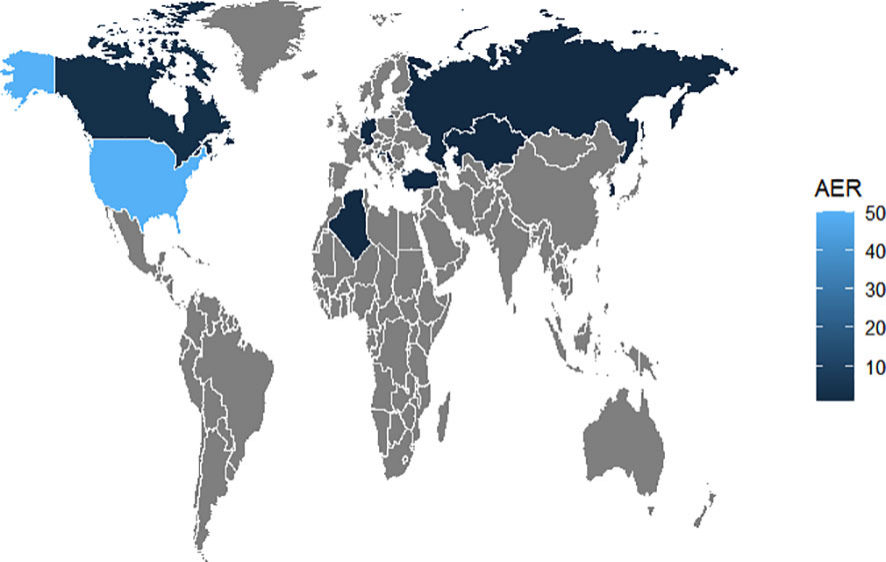
World map of the number of AEs reported.

The number of AE reports from 2005 to 2023 was extracted from FAERS totaling 10,698,312, from which a total of 74 adverse event reports for probiotic were screened, involving 285 PTs and a total of 15 SOCs. Among them, only 9 PTs and 2 SOCs all met the three algorithmic positive signals of ROR, PRR and EBGM. Annual case reports of AEs associated with probiotic preparations are shown in **Figure 2**, and the basic characteristics of these adverse reaction reports are shown in **Table 2**. Adverse reaction event reports associated with probiotic applications did not appear until 2005, and then showed a fluctuating growth until the number of reports peaked at 13 in 2022. However, in terms of the overall trend in the number of reports, the reporting of adverse reactions to probiotics remains at a low level or even some years with no adverse events reported. In terms of gender, the number of reported AEs is higher for male (N=41) than for female (N=17). In terms of age composition, the incidence of AEs was higher in the 70 to 79 age group (14.86%) than in other age groups. Interestingly, most of the reported data (67.57%) came from consumers, not medical professionals. In addition, the United States had the highest number of reports globally (84.79%). However, for reasons inherent to the FEARS data, the remaining countries providing AE reports are categorized as other. Categorizing the reports by country zones and making a world map, we can see that the U.S. region is the brightest blue, and that other countries reporting include a wide range of countries such as Canada, Germany, Russia, Kazakhstan, Algeria, Turkey, and South Korea (**Figure 3**). According to the analysis of FEARS data, non-serious adverse reactions (N=28) dominated the adverse reactions caused by probiotic preparations, while serious adverse reactions were mainly hospitalization, disability, life-threatening, death and other types of serious medical events. Apart from other types of serious medical events (54.55%), hospitalization (36.36%) was the most frequently reported serious ADR with 16 cases. Meanwhile, oral route of administration was the most common routes of administration, accounting for 51.35%.

**Table 2:** Clinical characteristics of reports with probiotic from the FAERS database (2005 Q1–2023 Q4).

Characteristics	Case Number	Case Proportion
Gender		
Male	17	22.97
Female	41	55.41
Unknown	16	21.62
Age		
<20	11	14.86
20~29	0	0
30~39	1	1.35
40~49	3	4.05
50~59	6	8.11
60~69	9	12.16
70~79	11	14.86
>80	3	4.05
Unknow	30	40.54
Reporter		
Consumer	50	67.57
Physician	7	9.46
Pharmacist	7	9.46
Other	2	2.70
Unknown	8	10.81
Reported Countries		
United States	50	67.57
Other	24	34.43
Outcomes		
Hospitalization	16	36.36
Life threatening	3	6.82
Disability	2	0.36
Death	1	2.27
Other serious	24	54.55
Application Route		
Oral	38	51.35
Other	36	48.65

### Signals detection associated with probiotic

#### Signals detection based on SOC levels

It was found that the ADRs induced by probiotic preparations mainly involved 15 SOCs. Among them, however, only two SOCs showed strong positives in the three algorithms. They were Gastrointestinal disorders (N=97, ROR=5.3, PRR=3.84, EBGM=3.84) and Hepatobiliary disorders (N=9, ROR=3.39, PRR=3.32, EBGM=3.32) (**Table 3**). In addition, SOCs that met two algorithms included Immune system disorders (ROR=2.14, PRR=2.11, EBGM=2.11). Ignoring the results of the EGBM algorithm, Immune system disorders also showed some signals of adverse reactions, suggesting a potential association of probiotic preparations causing Immune system-related adverse reactions. The results of our data analysis are consistent with the drug leaflet. As oral preparations, the most common adverse reactions to probiotics are primarily gastrointestinal. Moreover, Psychiatric disorders, Nervous system disorders, and Vascular disorders as never mentioned in the drug insert were also seen in the involved SOCs.

**Table 3:** The system organ class (SOC) for case reports in the FAERS database.

SOC	Case Reports	ROR (95% *CI*)	PRR (95% *CI*)	χ2	EBGM (EBGM05)
gastrointestinal disorders	97	5.3 (4.15, 6.77)	3.84 (3.28, 4.49)	223.12	3.84 (3.12)
hepatobiliary disorders	9	3.39 (1.75, 6.59)	3.32 (1.74, 6.34)	14.7	3.32 (2.10)
immune system disorders	7	2.14 (1.21, 4.53)	2.11 (1.22, 4.36)	4.15	2.11 (1.13)
metabolism and nutrition disorders	8	1.28 (0.64, 2.59)	1.28 (0.64, 2.54)	0.49	1.28 (0.71)
psychiatric disorders	19	1.16 (0.73, 1.84)	1.15 (0.75, 1.77)	0.37	1.15 (0.78)
infections and infestations	15	0.92 (0.55, 1.54)	0.92 (0.56, 1.5)	0.11	0.92 (0.6)
musculoskeletal and connective tissue disorders	14	0.85 (0.5, 1.45)	0.86 (0.52, 1.43)	0.36	0.86 (0.55)
general disorders and administration site conditions	40	0.73 (0.52, 1.02)	0.77 (0.57, 1.03)	3.35	0.77 (0.58)
nervous system disorders	18	0.7 (0.44, 1.13)	0.72 (0.46, 1.13)	2.14	0.72 (0.48)
renal and urinary disorders	4	0.67 (0.25, 1.8)	0.68 (0.26, 1.81)	0.63	0.68 (0.3)
vascular disorders	4	0.61 (0.23, 1.65)	0.62 (0.23, 1.65)	0.96	0.62 (0.27)
respiratory, thoracic and mediastinal disorders	9	0.61 (0.32, 1.19)	0.62 (0.32, 1.18)	2.15	0.62 (0.36)
investigations	11	0.6 (0.33, 1.1)	0.62 (0.34, 1.12)	2.76	0.62 (0.37)
injury, poisoning and procedural complications	17	0.6 (0.37, 0.98)	0.62 (0.39, 0.99)	4.31	0.62 (0.41)
skin and subcutaneous tissue disorders	6	0.36 (0.16, 0.82)	0.38 (0.17, 0.83)	6.52	0.38 (0.19)

SOC, system organ classes; ROR, reporting odds ratio; PRR, proportional reporting ratio ; 95%*CI*, 95% confidence interval; *χ2*, chi-squared; EBGM, empirical Bayesian geometric mean; EBGM05, the lower bound of 95% *CI.*

#### Signals detection based on PT levels

The ROR algorithm is one of the most commonly used algorithms, and we ranked the preferred term (PT) that met the criteria of the three algorithms in descending order of signal strength (ROR [95%*CI*] values) to obtain all SOCs, with Gastrointestinal pain having the highest ROR (95%*CI*) signal strength. In addition, some unexpected adverse reactions such as Agitation and Anxiety were included (**Table 4**). Among them, the three PTs with the highest number of reported cases were Abdominal discomfort (N= 11), Vomiting (N= 9) and Abdominal distension (N= 8). Notably, in terms of risk of AEs, Gastrointestinal pain was the highest (ROR=77.76, PRR=76.69, EBGM=76.63), followed by Hypophagia (ROR=24.13, PRR=23.88, EBGM=28.88) and Flatulence (ROR=23.75, PRR=23.28, EBGM=23.27).

**Table 4:** The preferred terms (PT) for case reports in the FAERS database that met the three algorithmic criteria.

PT	SOC	Case Reports	ROR (95% *CI*)	PRR (95% *CI*)	χ2	EBGM (EBGM05)
gastrointestinal pain	gastrointestinal disorders	4	77.76 (28.97, 208.73)	76.69 (28.78, 204.34)	298.62	76.63 (33.54)
hypophagia	metabolism and nutrition disorders	3	24.13 (7.73, 75.27)	23.88 (7.81, 72.98)	65.79	23.88 (9.22)
flatulence	gastrointestinal disorders	6	23.75 (10.58, 53.34)	23.28 (10.63, 50.99)	128	23.27 (11.83)
abdominal distension	gastrointestinal disorders	8	16.68 (8.26, 33.7)	16.24 (8.18, 32.25)	114.62	16.24 (9.02)
abdominal discomfort	gastrointestinal disorders	11	13.7 (7.5, 25.04)	13.21 (7.34, 23.78)	124.51	13.21 (7.98)
agitation	psychiatric disorders	4	12.48 (4.65, 33.48)	12.32 (4.62, 32.83)	41.63	12.32 (5.39)
abdominal pain upper	gastrointestinal disorders	5	5.07 (2.1, 12.29)	5 (2.11, 11.84)	16.07	5 (2.39)
vomiting	gastrointestinal disorders	9	4.2 (2.16, 8.15)	4.1 (2.15, 7.83)	21.22	4.1 (2.35)
anxiety	psychiatric disorders	6	4.1 (1.83, 9.21)	4.04 (1.84, 8.85)	13.77	4.04 (2.05)

PT, preferred terms; SOC, system organ classes; ROR, reporting odds ratio; PRR, proportional reporting ratio; 95%*CI*, 95% confidence interval; *χ2*, chi-squared; EBGM, empirical Bayesian geometric mean; EBGM05, the lower bound of 95% *CI.*

**Figure 4:**
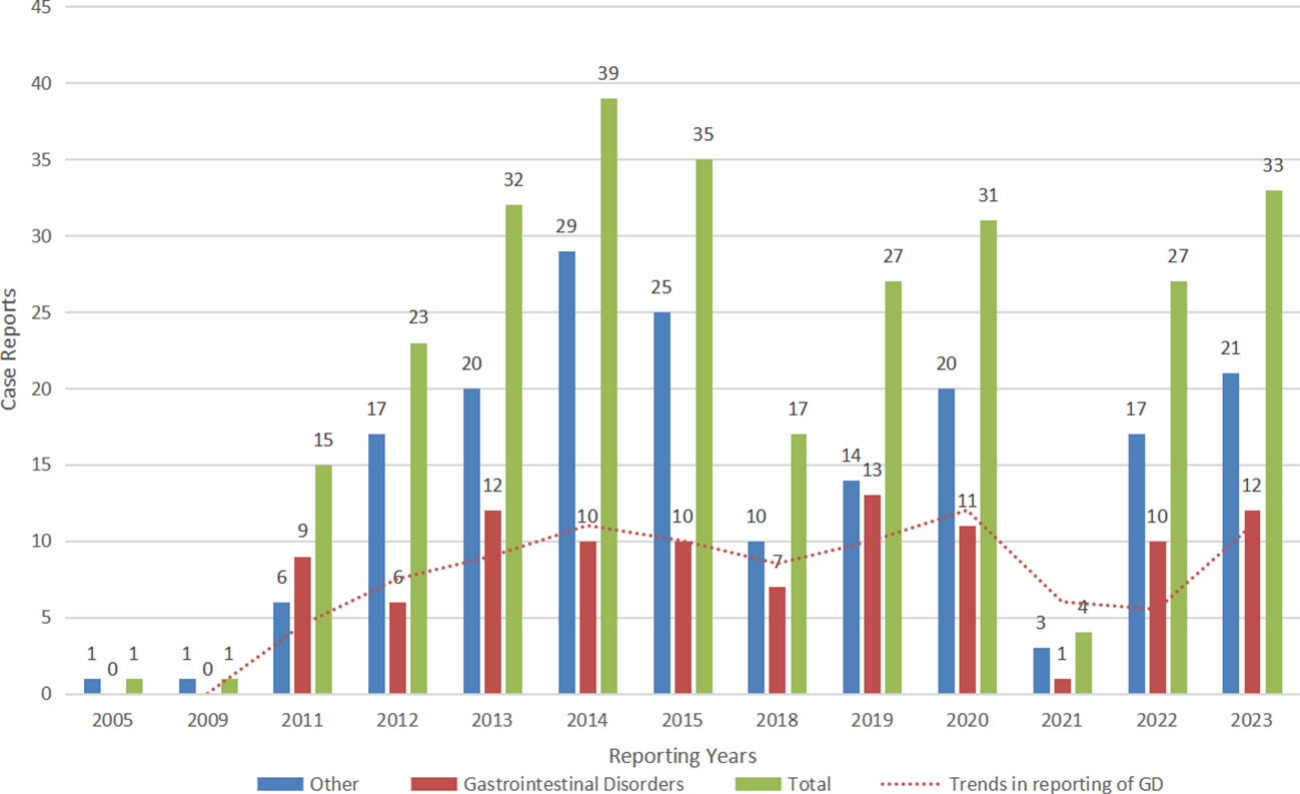
The bar graph shows the number of case reports of probiotic adverse events in the FAERS database from 2005 through 2023. Gastrointestinal disorders: the number of case reports of gastrointestinal disorders associated with probiotic per year; other adverse events: the number of case reports of other adverse events associated with probiotic per year (with gastrointestinal disorders removed); total: the total number of adverse events associated with probiotic per year.

The results of this study showed that PTs that met all the algorithm criteria were mainly associated with Gastrointestinal disorders (2/3). Therefore, we reintegrated the above PTs with R language and redefined them as Gastrointestinal disorders or Others. Subsequently, we plotted year-based bar graph of changes in the number of probiotic AE reports to demonstrate reporting trends for both types of AEs. As shown in **Figure 4**, the number of Gastrointestinal disorders submitted to the FAERS database has been reported since 2011 and has been at a level of 10 cases per year and fluctuating up and down, peaking at 13 cases in 2019. Notably, the overall number of probiotic adverse reactions reported in 2021 decreased significantly, and thus the number of gastrointestinal adverse reactions decreased dramatically downward.

### Subgroups analysis

#### Signals detection based on SOC levels

### Gender

In this study, AEs were also analyzed as subgroups base on SOC level according to gender. The results of the identification of adverse reaction signals for probiotic application based on gender grouping are shown in **Table 5**. For the male group, there were two SOCs for which all three algorithms suggested strong positive signals, namely Gastrointestinal disorders (N=30, ROR=6.96, PRR=4.64, EBGM=4.64) and Metabolism and nutrition disorders (N=6, ROR=3.21, PRR=3.04, EBGM=3.04). As for the female group, also two SOCs met all the algorithms, namely Hepatobiliary disorders (N=9, ROR=6.44, PRR=6.14, EBGM=6.14) and Gastrointestinal disorders (N=51, ROR=4.39, PRR=3.34, EBGM=3.34). The results suggest that gender differences have no significant effect on the occurrence of gastrointestinal dysfunction. However, due to the wide disparity in gender reporting of certain SOCs, such as Metabolism and nutrition disorders and Hepatobiliary disorders seen in only one of the genders, we cannot readily speculate on the potential influence of gender on such AEs.

**Table 5:** The system organ class (SOC) for case reports in the FAERS database based on gender subgroups.

SOC	Male					Female				
	Case Reports	ROR (95%*CI*)	PRR (95%*CI*)	*χ2*	EBGM (EBGM05)	Case Reports	ROR (95%*CI*)	PRR (95%*CI*)	*χ2*	EBGM (EBGM05)
gastrointestinal disorders	30	6.96 (4.4, 11)	4.64 (3.53, 6.11)	93.4	4.64 (3.16)	51	4.39 (3.16, 6.11)	3.34 (2.64, 4.23)	92.27	3.34 (2.54)
general disorders and administration site conditions	9	0.64 (0.32, 1.28)	0.68 (0.37, 1.25)	1.64	0.68 (0.38)	31	1.01 (0.68, 1.49)	1.01 (0.74, 1.38)	0	1.01 (0.73)
infections and infestations	7	1.63 (0.75, 3.55)	1.57 (0.78, 3.18)	1.56	1.57 (0.82)	7	0.72 (0.34, 1.55)	0.74 (0.36, 1.53)	0.7	0.74 (0.39)
musculoskeletal and connective tissue disorders	4	1.08 (0.4, 2.96)	1.08 (0.41, 2.82)	0.02	1.08 (0.46)	9	0.82 (0.42, 1.61)	0.83 (0.44, 1.55)	0.34	0.83 (0.47)
nervous system disorders	7	1.06 (0.49, 2.31)	1.06 (0.52, 2.15)	0.02	1.06 (0.55)	9	0.57 (0.29, 1.12)	0.6 (0.32, 1.12)	2.7	0.6 (0.34)
metabolism and nutrition disorders	6	3.21 (1.39, 7.38)	3.04 (1.42, 6.53)	8.41	3.04 (2.15)	–	–	–	–	–
hepatobiliary disorders	–	–	–	–	–	9	6.44 (3.29, 12.61)	6.14 (3.28, 11.5)	39.08	6.14 (3.5)
immune system disorders	–	–	–	–	–	4	1.96 (0.73, 5.29)	1.94 (0.74, 5.07)	1.84	1.94 (0.85)
psychiatric disorders	–	–	–	–	–	8	0.87 (0.43, 1.78)	0.88 (0.45, 1.71)	0.14	0.88 (0.49)
respiratory, thoracic and mediastinal disorders	–	–	–	–	–	7	0.82 (0.39, 1.75)	0.83 (0.4, 1.71)	0.26	0.83 (0.44)
investigations	–	–	–	–	–	7	0.71 (0.33, 1.52)	0.72 (0.35, 1.49)	0.78	0.72 (0.38)
injury, poisoning and procedural complications	–	–	–	–	–	7	0.43 (0.2, 0.92)	0.45 (0.22, 0.93)	5.07	0.45 (0.24)
skin and subcutaneous tissue disorders	–	–	–	–	–	4	0.38 (0.14, 1.03)	0.4 (0.15, 1.05)	3.89	0.4 (0.17)

SOC, system organ classes; ROR, reporting odds ratio; PRR, proportional reporting ratio; 95%*CI*, 95% confidence interval; *χ2*, chi-squared; EBGM, empirical Bayesian geometric mean; EBGM05, the lower bound of 95% *CI.*

### Age

We divided and reclassified the age of AE-reporting patients into two groups (minor group and adult group) with a cut-off of 18 years, followed by subgroup analyses to identify signals of adverse effects (**Table 6**). Four SOCs were mainly involved in the minor group, while 10 SOCs were involved in the underage group. For the minor group, the only 1 SOC that met all three algorithms suggesting strong positivity was Gastrointestinal disorders (ROR=9.85, PRR=5.84, EBGM=5.84), whereas two SOCs met all the algorithmic requirements in the adult group, which were Hepatobiliary disorders (ROR=5.24, PRR=5, EBGM=5) and Gastrointestinal disorders (ROR=4.37, PRR=4.33, EBGM=4.32). And ignoring the results of EBGM, metabolism and nutrition disorders (ROR=4.28, PRR=3.96, EBGM=3.96) were more likely to be associated with the minor group.

**Table 6:** The system organ class for case reports in the FAERS database based on age subgroups.

SOC	>18					<=18				
	Case Reports	ROR (95%*CI*)	PRR (95%*CI*)	*χ2*	EBGM (EBGM05)	Case Reports	ROR (95%*CI*)	PRR (95%*CI*)	*χ2*	EBGM (EBGM05)
gastrointestinal disorders	45	4.37 (3.07, 6.21)	3.33 (2.63, 4.21)	80.69	3.32 (2.48)	19	9.85 (5.36, 18.08)	5.84 (4.19, 8.15)	82.69	5.84 (3.51)
general disorders and administration site conditions	28	1.17 (0.77, 1.76)	1.14 (0.82, 1.59)	0.55	1.14 (0.8)	4	0.66 (0.24, 1.85)	0.69 (0.27, 1.77)	0.63	0.69 (0.29)
hepatobiliary disorders	8	5.24 (2.57, 10.69)	5 (2.57, 9.74)	25.92	5 (2.76)	–	–	–	–	–
immune system disorders	4	2.55 (0.94, 6.88)	2.5 (0.96, 6.53)	3.65	2.5 (1.09)	–	–	–	–	–
musculoskeletal and connective tissue disorders	10	1.15 (0.6, 2.18)	1.14 (0.62, 2.09)	0.18	1.14 (0.66)	–	–	–	–	–
respiratory, thoracic and mediastinal disorders	8	1 (0.49, 2.05)	1 (0.51, 1.95)	0	1 (0.55)	–	–	–	–	–
psychiatric disorders	6	0.75 (0.33, 1.7)	0.76 (0.35, 1.66)	0.47	0.76 (0.38)	–	–	–	–	–
nervous system disorders	10	0.75 (0.39, 1.42)	0.77 (0.42, 1.41)	0.79	0.77 (0.45)	–	–	–	–	–
investigations	6	0.61 (0.27, 1.38)	0.63 (0.29, 1.38)	1.43	0.63 (0.32)	–	–	–	–	–
injury, poisoning and procedural complications	6	0.46 (0.2, 1.04)	0.48 (0.22, 1.05)	3.62	0.48 (0.24)	–	–	–	–	–
metabolism and nutrition disorders	–	–	–	–	–	4	4.28 (1.53, 11.98)	3.96 (1.55, 10.15)	9.08	3.96 (1.67)
infections and infestations	–	–	–	–	–	4	1.57 (0.56, 4.4)	1.52 (0.59, 3.89)	0.75	1.52 (0.64)

SOC, system organ classes; ROR, reporting odds ratio; PRR, proportional reporting ratio; 95%*CI*, 95% confidence interval; *χ2*, chi-squared; EBGM, empirical Bayesian geometric mean; EBGM05, the lower bound of 95% *CI.*

### Signals detection based on PTs

**Figure 5:**
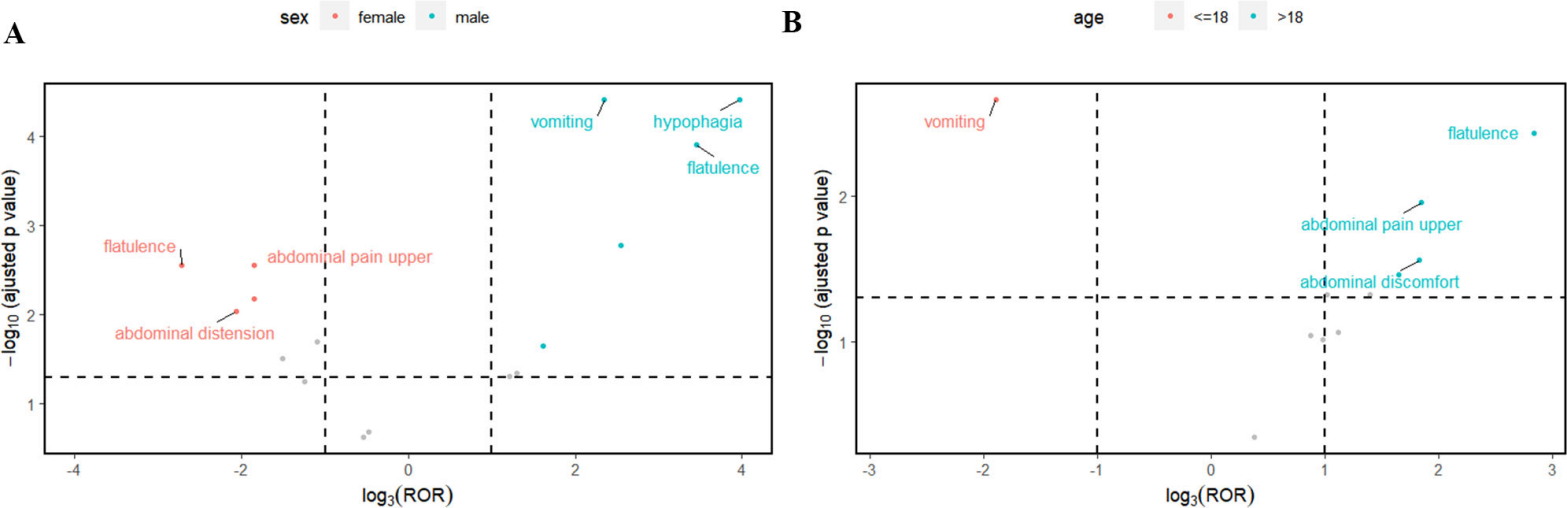
The top 3 preferred terms (PT) for case reports in the FAERS database that met the three algorithmic criteria based on gender **(A)** or age **(B)** subgroups.

Subsequently, this study also performed subgroups analysis by gender or age to identify PT signals that met the criteria of the three algorithms (**Tables 7**, **8**). In the male group, the total number of PTs suggestive of positivity for all 3 algorithms was 4, with Abdominal pain upper (N=5) being the highest in terms of number of events reported. In the female group, on the other hand, 5 PTs fulfilled all three algorithms suggestive of positivity, of which the top PTs were Vomiting (N= 6). In addition, asthenia unexpectedly appeared in the male group and suggested a strong adverse signal (N=3, ROR=5.9, PRR=5.71, EBGM=5.71) (**Table 7**). The results of the age subgroup analysis informed that only Vomiting prompted strong positivity in the minor group (N=4, ROR=8, PRR=7.34, EBGM=7.33), whereas in the adult group four PTs were strongly positive in all three algorithms, namely Flatulence (N=3, ROR=22.6, PRR=22.15, EBGM=22.15), Abdominal discomfort (N=4, ROR=7.64, PRR=7.45, EBGM=7.45), abdominal pain upper (N=3, ROR=7.48, PRR=7.35, EBGM=7.35) and constipation (N=3, ROR=6.14, PRR=6.04, EBGM=6.04) (**Table 8**). We then visualized the above results in a volcano diagram (**Figure 5**).

**Table 7:** The system organ class (SOC) for case reports in the FAERS database based on gender subgroups.

SOC	PT	Male					Female				
		Case Reports	ROR (95%*CI*)	PRR (95%*CI*)	*χ2*	EBGM (EBGM05)	Case Reports	ROR (95%*CI*)	PRR (95%*CI*)	*χ2*	EBGM (EBGM05)
gastrointestinal disorders	flatulence	3	44.31 (13.97, 140.57)	42.62 (13.95, 130.26)	122.01	42.61 (16.22)	3	19.89 (6.35, 62.31)	19.54 (6.39, 59.72)	52.82	19.54 (7.51)
gastrointestinal disorders	abdominal discomfort	3	16.44 (5.18, 52.14)	15.84 (5.18, 48.41)	41.79	15.83 (6.03)	4	7.6 (2.82, 20.5)	7.44 (2.85, 19.44)	22.37	7.44 (3.24)
gastrointestinal disorders	vomiting	6	13.15 (5.72, 30.26)	12.2 (5.68, 26.2)	62.11	12.2 (6.08)	–	–	–	–	–
general disorders and administration site conditions	asthenia	3	5.9 (1.86, 18.72)	5.71 (1.87, 17.45)	11.74	5.71 (2.17)	–	–	–	–	–
metabolism and nutrition disorders	hypophagia	3	78.21 (24.65, 248.14)	75.2 (24.61, 229.83)	219.61	75.15 (28.6)	–	–	–	–	–
gastrointestinal disorders	abdominal distension	–	–	–	–	–	3	9.69 (3.09, 30.36)	9.53 (3.12, 29.13)	22.95	9.53 (3.66)
gastrointestinal disorders	abdominal pain upper	–	–	–	–	–	5	7.65 (3.14, 18.63)	7.45 (3.15, 17.65)	28.01	7.45 (3.54)

PT, preferred terms; SOC, system organ classes; ROR, reporting odds ratio; PRR, proportional reporting ratio; 95%*CI*, 95% confidence interval; *χ2*, chi-squared; EBGM, empirical Bayesian geometric mean; EBGM05, the lower bound of 95% *CI.*

**Table 8:** The system organ class (SOC) for case reports in the FAERS database based on age subgroups.

SOC	PT	>18					<=18				
		Case Reports	ROR (95%*CI*)	PRR (95%*CI*)	*χ2*	EBGM (EBGM05)	Case Reports	ROR (95%*CI*)	PRR (95%*CI*)	*χ2*	EBGM (EBGM05)
gastrointestinal disorders	flatulence	3	22.6 (7.2, 70.92)	22.15 (7.25, 67.7)	60.64	22.15 (8.51)	–	–	–	–	–
	abdominal pain upper	4	7.64 (2.83, 20.63)	7.45 (2.85, 19.46)	22.43	7.45 (3.24)	–	–	–	–	–
	constipation	3	6.14 (1.96, 19.27)	6.04 (1.98, 18.46)	12.65	6.04 (2.32)	–	–	–	–	–
	abdominal discomfort	3	7.48 (2.38, 23.47)	7.35 (2.4, 22.46)	16.49	7.35 (2.82)	–	–	–	–	–
	vomiting	–	–	–	–	–	4	8 (2.86, 22.43)	7.34 (2.86, 18.81)	22.17	7.33 (3.1)

PT, preferred terms; SOC, system organ classes; ROR, reporting odds ratio; PRR, proportional reporting ratio; 95%*CI*, 95% confidence interval; *χ2*, chi-squared; EBGM, empirical Bayesian geometric mean; EBGM05, the lower bound of 95% *CI.*

## Discussion

Calls for attention to probiotic AEs are increasing, and some clinical studies have reported on these AEs (Zawistowska-Rojek and Tyski, 2018; Suez et al., 2019); however, more comprehensive studies are still lacking. This study was derived from an in-depth analysis of AE reports on probiotics in the FAERS database, with the aim of providing real-world data to guide the clinical application of probiotics. Although there have been researchers who have investigated the risk-benefit of probiotics in certain types of patients based on reports from the FAERs database of AEs (Bennett, 2016). However, to our knowledge, this is the first pharmacovigilance analysis of probiotics from multiple perspectives.

### Demographic characteristics of probiotic adverse events

We have extracted over 10 million AE records from the FEARS database for all FDA marketed drugs or related products. Among them, there were 74 AEs related to oral probiotic products. The results of the study reflect a very low number of probiotic-associated adverse reactions and also indicate that the safety profile of probiotic preparations is generally favorable, in line with existing public conclusions. In addition, we cannot ignore that some probiotics are defined as dietary supplements, which tends to reduce the urgency to report probiotic-related AEs (Hempel et al., 2011; Merenstein et al., 2023). This study analyzed the reported demographic characteristics of the probiotic AEs from FAERS database. And, the results showed that in terms of the gender composition ratio of patients, it appeared that a higher percentage of females (55.41%) reported than males (22.97%). This may be due to the fact that women are more inclined to initiate reporting of adverse events (Jiang et al., 2024). In addition, there may be some gender differences in probiotic-mediated effects, and gut flora can be influenced by the hormonal environment (Snigdha et al., 2022). The concept of the “microgenderome” reflects the involvement of the gut microbiota in the host’s sex hormone excretion and cycling processes (Yoon and Kim, 2021). Much evidence reveals gender differences including immunologic, genetic, hormonal, and environmental factors and contributes to gender-specific responses and outcomes to medications (Flanagan et al., 2017). Thus, an imbalance in the proportion of probiotic AEs occurring in different gender populations seems to be explained. In terms of age composition, patients reporting probiotic AEs were predominantly middle-aged and elder people over 50 years of age, accounting for approximately 39.19%. The current study concluded that probiotic AEs are more likely to occur in older adults or critically ill infants (Didari et al., 2014). However, it is noteworthy that a large portion of the data (40.54%) lacked age-specific details, limiting our ability to accurately analyze the occurrence of true age-specific adverse events. We advocate for future researchers or reporters in this field to provide more complete age data, which would facilitate the exploration of age-based differences in drug response. Surprisingly, over 60% of the percentage of AE reports came from consumers themselves rather than medical or pharmacy professionals. Although, this shows the positivity and initiative of consumers in reporting AEs, it also shows the negligence of probiotic AEs by the professionals in the field. In addition, the United States contributed the majority of the reported data (67.57%), with only a small portion of the AE reports dispersed among a few countries. The countries where AE reports have occurred are concentrated in the North American region as well as in Europe. There may be a correlation between the importance a country’s population places on the safety of drug application and its level of economic development.

### Signal recognition of probiotic adverse events based on global data

Gastrointestinal disorders, Hepatobiliary disorders, and Immune system disorders are signs of adverse reactions unique to probiotic. The conclusions of this study are consistent with the drug insert, which states that as an oral preparation, the most common adverse reactions to probiotics are mainly gastrointestinal reactions. A systematic review exposes a severe allergic reaction (severe urticaria) associated with probiotics in a patient with cystic fibrosis and gastrointestinal disturbances including vomiting and diarrhea in two pediatric patients (Coffey et al., 2020). Another report summarizes the adverse effects seen in patients with Ulcerative colitis in a clinical randomized controlled trial of probiotic VSL#3 versus Fecal microbiota transplantation (Dang et al., 2020). Although, there were no statistically significant serious adverse events with probiotics VSL#3 (*P*=0.84), however, gastrointestinal disturbances such as abdominal distension and mouth odor may still occur in some patients. In addition, certain adverse reactions never mentioned in the drug insert, such as psychiatric disorders, neurological disorders and vascular disorders, do not present significant positive signals for the time being, but still, such signals should be highly noticed and monitored in high-risk groups.

At the PT level, Symptoms of gastrointestinal disorders such as abdominal discomfort, vomiting and abdominal distension had the highest incidence. Also, this study identified AEs not mentioned in the medication leaflet, such as psychiatric disorders like agitation and anxiety. The gut microbiota can influence host behavior and activity through the “microbe-gut-brain axis” (Nikolova et al., 2021). The intake of probiotics regulates the balance of the intestinal flora and affects the brain function of the host by stimulating the vagus nerve to establish a direct connection and influence changes in the release of neurotransmitters, hormones and other metabolites (Kleiman et al., 2017). After four weeks of oral probiotic administration to depressed patients, a relative increase in β-diversity and abundance of butyric acid bacteria such as *Ruminococcus gauvreauii* and *Coprococcus 3* was observed in the gut (Reininghaus et al., 2020). Short-chain fatty acids such as butyric acid are important in maintaining the integrity of the intestinal barrier and contribute to suprachiasmatic brain-derived neurotrophic factor after entering the bloodstream (Mörkl et al., 2020). However, Ng et al. (2023). noted a lack of reporting of adverse effects and good long-term data tracking in clinical controlled studies of probiotics for the treatment of depressed patients. Several studies also have demonstrated that transplantation of fecal microbiota leads to the development of altered psychiatric behavior and physiological characteristics in mice (Li et al., 2019; Zhu et al., 2020). Despite the benefits of probiotics in normal people, we cannot completely rule out the possibility that probiotics may cause psychiatric abnormalities in the host via the microbe-gut-brain axis. More research is needed to explore the effects and potential mechanisms of probiotics on host mental and behavioral activities. The novel findings of this study also indicate that quantitative signal detection technology has a promising future in the monitoring of adverse drug events to scientifically tap potential risk signals.

### Signal recognition of probiotic adverse events based on subgroup analysis

There is no denying that there is a correlation between gender differences and factors such as age and the occurrence of diseases, including gastrointestinal disorders (Lee, 1962), liver diseases (Yip et al., 2022), and cancer (Stjernfelt et al., 2020). In this study, probiotic AEs from the FAERS database were analyzed in subgroups according to gender and age. In this case, gender differences were sex differences in physiologic categories, while age was grouped by reporting data as adult or not. At the SOC level, the results of this study show that gastrointestinal disorders were plausible signal of adverse effects in both males and females. Also, age subgroup analysis suggested the positive adverse signals of gastrointestinal disorders in different age groups. We concluded that the effect of gender differences or age on gastrointestinal disorders were not significant. In addition, the male or minor group was more susceptible to Metabolism and nutrition disorders; whereas, females or adult groups was more prone in Hepatobiliary disorders. At the PT level, results of the gender and age subgroup analyses suggested that several PTs were significant signals of AEs. Among them, we unexpectedly found that asthenia was more likely to occur in the male group. This adverse reaction, which is not listed in the drug insert, requires prompt attention and appropriate management. The probiotic preparations involved in this study included topical medications in addition to oral administration, of which topical probiotics for the female reproductive tract were among them. However, the cases included in the subgroup analyses suffered from a significant gender imbalance, and the lack of explicit gender labeling in some of the reports may have contributed to the controversial gender-differentiated results. Therefore, we should be cautious about the above conclusions and recommend better gender labeling, larger samples, and SOC-based clinical studies and monitoring of adverse effects. In addition, it should be noted that due to the small number of AEs in this study, the results obtained from subgroup analyses may be subject to some error due to insufficient numbers of cases rather than true biological differences.

### Concomitant medications and adverse reactions

Studies have reported incidents related to interactions between prescription drugs and certain food supplements containing bioactive ingredients (de Boer et al., 2015). It was also pointed out that the different pharmacokinetic characteristics of different drugs should be carefully considered, and that drug-drug interactions and the resulting adverse drug reactions should be avoided, which would be more conducive to improving patient compliance (Bellosta and Corsini, 2012). A Korean Adverse Drug Reaction Reporting study analyzed parenteral nutrition-related adverse reactions and also analyzed the effects of common concomitant medications on parenteral nutrition-related adverse reactions. The study found that common co-administered medications (e.g., fentanyl, ketorolac, and tramadol) were analyzed by subgroups and still supported that nausea and vomiting p parenteral nutrition itself caused (Eum et al., 2019). Unfortunately, our study was unable to analyze the effect of coadministration on AE due to the lack of information on coadministration in the AE data. However, several studies have examined the interaction between probiotics and co-medication. Probiotics are commonly used to prevent antibiotic-induced dysbiosis of the intestinal flora, and supplementation with probiotics during antibiotic therapy does not affect the diversity index of the intestinal microbiome (Éliás et al., 2023). In addition, there is weak evidence that probiotics may enhance the efficacy of immune checkpoint inhibitors (Li et al., 2023). In animal studies, intake of various probiotics was found to interfere with monosodium glutamate-induced obesity and phagocytic pro-inflammatory polarization in rats (Rudyk et al., 2023). Although there are several lines of evidence supporting the positive effects of probiotics when used in conjunction with other drugs, there is still a gap in the research on the possible adverse effects of concomitant medications.

### Limitations

This study evaluates the safety of probiotics from different perspectives, and to a certain extent provides a more scientific and reasonable application guidance. However, it is undeniable that there are still some limitations. First, the data source of the FEARS database relies mainly on spontaneous reports from individuals, the vast majority of which come from non-medical professionals, which may lead to reporting bias and incomplete information. Second, the number of reported contributions is significantly unbalanced across countries and regions, which may be subject to sampling bias. Third, reporting is still subject to a lack of reporting of potential confounders, such as concomitant diseases or coadministration of medications, leading to biased results. Finally, the large variation in different probiotic strains and content in probiotic preparations constrains stratified analyses based on differences in strain composition. Therefore, future researchers in the field of probiotic transformation and application should look at probiotic strains and content as points of observation, and conduct long-term clinical trials and monitoring of adverse reactions based on factors such as age and gender. At the same time, co-reporting of concomitant diseases and concomitant medications in cases should be strengthened in order to enhance the rationality of data reported by AE.

## Conclusion

The findings of this study, based on the FAERS system, continue to support the overall safety of probiotic preparations. Among them, gastrointestinal adverse reactions and immune system disorders remain common probiotic adverse reactions. And psychoneurological disorders represented by agitation and anxiety are potential new signals of probiotic adverse reactions. Meanwhile, males and minors were more likely to have metabolic and nutritional disorders; females and adult groups were more likely to have hepatobiliary system adverse reactions. This study is the first to systematically analyze the adverse reactions reported by individuals to probiotic preparations, and despite the limitations of the data, the results have important implications for future translational research and long-term safety monitoring of probiotics.

## Data Availability

The datasets presented in this study can be found in online repositories. The names of the repository/repositories and accession number(s) can be found below: DOI 10.5072/zenodo.68617.
