# Supplementary material for: A pharmacovigilance study on probiotic preparations based on the FDA Adverse Event Reporting System from 2005 to 2023
Source: Front Cell Infect Microbiol. 2025 May 13;15:1455735. doi: 10.3389/fcimb.2025.1455735 (PMC12106448; doi:10.3389/fcimb.2025.1455735)
Supplement: Supplementary file 1 [file Table1.doc]

**Table S1. Fourfold table of disproportionality method.**

| Drugs | Target AEs reported | Other AEs reported | Summation |
| --- | --- | --- | --- |
| Suspect drugs | a | b | a+b |
| Other drugs | c | d | c+d |
| Summation | a+c | b+d | a+b+c+d |

AEs: adverse events.
